# Flavonoid Nobiletin Attenuates Cyclophosphamide-Induced Cystitis in Mice through Mechanisms That Involve Inhibition of IL-1β Induced Connexin 43 Upregulation and Gap Junction Communication in Urothelial Cells

**DOI:** 10.3390/ijms23095037

**Published:** 2022-05-01

**Authors:** Jin Kono, Masakatsu Ueda, Atsushi Sengiku, Sylvia O. Suadicani, Je Tae Woo, Takashi Kobayashi, Osamu Ogawa, Hiromitsu Negoro

**Affiliations:** 1 Department of Urology, Kyoto University Graduate School of Medicine, Kyoto 606-8507, Japan; konojin@kuhp.kyoto-u.ac.jp (J.K.); adeu@kuhp.kyoto-u.ac.jp (M.U.); sengiku@kuhp.kyoto-u.ac.jp (A.S.); selecao@kuhp.kyoto-u.ac.jp (T.K.); ogawao@kuhp.kyoto-u.ac.jp (O.O.); 2 Department of Urology, Shizuoka General Hospital, Shizuoka 420-8527, Japan; 3 Sengiku Urology Clinic, Shiga 524-0045, Japan; 4 Department of Urology, Albert Einstein College of Medicine, Bronx, NY 10461, USA; sylvia.suadicani@einsteinmed.org; 5 Department of Biological Chemistry, College of Bioscience and Biotechnology, Chubu University, Kasugai 487-8501, Japan; woojetae@gmail.com; 6 Department of Urology, Faculty of Medicine, University of Tsukuba, Ibaraki 305-8575, Japan

**Keywords:** bladder inflammatory diseases, urothelium, connexin 43, gap junction, nobiletin

## Abstract

Bladder inflammatory diseases cause various urinary symptoms, such as urinary frequency and painful urination, that impair quality of life. In this study, we used a mouse model of cyclophosphamide (CYP)-induced bladder inflammation and immortalized human urothelial (TRT-HU1) cells to explore the preventive potential of nobiletin (NOB), a polymethoxylated flavone enriched in citrus fruit peel, and investigate its mechanism of action in the bladder. Prophylaxis with PMF90 (60% NOB) attenuated the development of bladder inflammation and urinary symptoms in CYP-treated mice. PMF90 also reduced the upregulation of connexin 43 (Cx43), a major component of gap junction channels, in the bladder mucosa of CYP-treated mice. Stimulation of TRT-HU1 cells with the pro-inflammatory cytokine IL-1β increased Cx43 mRNA and protein expression and enhanced gap junction coupling—responses that were prevented by pre-treatment with NOB. In urothelium-specific Cx43 knockout (uCx43KO) mice, macroscopic signs of bladder inflammation and changes in voiding behavior induced by CYP treatment were significantly attenuated when compared to controls. These findings indicate the participation of urothelial Cx43 in the development of bladder inflammation and urinary symptoms in CYP-treated mice and provide pre-clinical evidence for the preventive potential of NOB through its anti-inflammatory effects on IL-1β signaling and urothelial Cx43 expression.

## 1. Introduction

Chronic inflammatory bladder diseases, such as interstitial cystitis (IC) and nonbacterial cystitis (e.g., allergic), cause debilitating urinary and pelvic pain symptoms that significantly impair quality of life. These conditions remain a challenge for health care providers and scientists, given the inherent difficulties in their diagnosis, the lack of specific and effective therapies, and their recognized multifactorial nature [1]. Current treatments include behavioral modifications, use of anti-allergic and anti-inflammatory drugs, dimethyl sulfoxide solution, pain modulators, anticholinergics and drugs that protect the bladder mucosa, or bladder distension, neuromodulation, and surgical interventions [2,3,4]. However, the efficacy of surgical treatments, such as fulguration of the Hunner lesions [5,6,7,8] or hydrodistension [9,10,11,12,13], are not long-lasting and repeated treatments are often required. Other clinical trials, including intravesical injections of hyaluronic acid, botulinum toxin A (Botox A), triamcinolone, resiniferatoxin, and platelet-rich plasma, or preclinical trials of Botox A plus shockwave, have been conducted, but these have not yet become established treatments [14,15]. There is, therefore, an unmet and critical need for less invasive, more efficient, and specific preventions and treatments for such recurrent and resistant diseases.

In recent years, polymethoxyflavonoids (PMFs) such as nobiletin (NOB) and tangeretin that are found in high concentrations in Shikuwasa (*Citrus depressa*) and in other citrus fruit peel extracts have been attracting increasing attention for their several bioactive properties. Of these properties, the PMFs’ anti-inflammatory, antioxidant, and anti-allergic actions [16,17,18,19,20,21] make them ideal candidates to examine as potential novel drugs for treatment of bladder inflammatory conditions.

Animal models of bladder inflammation, such as those induced by cyclophosphamide (CYP), acetic acid, and hydrogen peroxide [22,23,24,25,26,27], have been widely employed in pre-clinical and mechanistic studies of IC. We previously reported that the increased urinary frequency and detrusor smooth contractions observed in CYP-treated mice were attenuated by treatment with gap junction blockers and were associated with a marked upregulation of the gap junction protein connexin 43 (Cx43) in the urinary bladder tissue [28]. A recent in vivo study, conducted to assess the effects of NOB treatment on bovine oocyte maturation and subsequent embryo development, have also shown that the expression of several genes, including *GJA1*, which encodes Cx43, are altered in the cumulus cells from oocytes matured in the presence of NOB [29]. In the bladder, Cx43 is expressed not only in the detrusor smooth muscle layer but also in the urothelium and has been shown to be an essential player in mechanisms that regulate functional bladder capacity [28,30,31,32]. The role that urothelial dysfunction plays in bladder inflammation and in the emergence of urinary and pelvic pain symptoms has been the focus of intense research [33,34], but the involvement of urothelial Cx43 in the underlying mechanisms is still unknown.

In the studies that we describe here, we used the animal model of CYP-induced cystitis in mice to examine both the prophylactic effect of NOB and tangeretin on bladder inflammation and associated urinary symptoms and investigated whether urothelial Cx43 is involved in the mechanisms underlying the action of these PMFs on the bladder.

## 2. Results

### 2.1. Prophylactic Treatment with PMF90 Improved Urinary Symptoms in the CYP-Induced Cystitis Mouse Model

**Figure 1:**
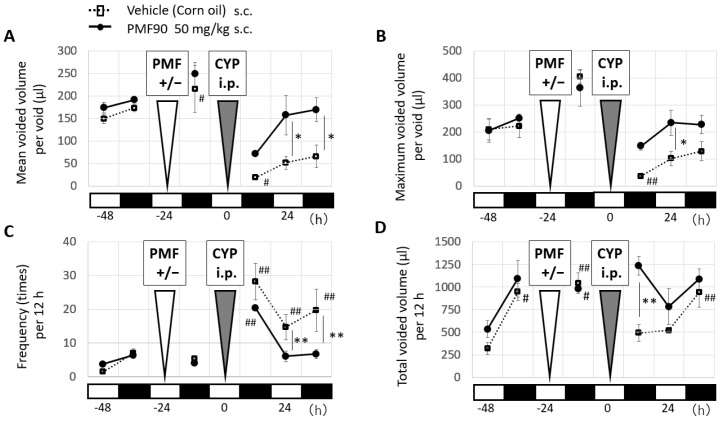
Effect of prophylactic treatment with PMF90 on the voiding behavior of mice following cyclophosphamide (CYP)-induced cystitis. The light and dark grey triangles show the time of PMF90 [50 mg/kg subcutaneous (s.c.), indicated as “+”, or vehicle, indicated as “-”] and CYP [150 mg/kg intraperitoneal (i.p.)] administration, respectively. The white and black squares along the X-axis indicate the light and dark periods, respectively, of the 12 h light cycle (9:00 am to 9:00 pm). Voiding behavior was assessed using the automated void stain on paper (aVSOP) method every 12 h, before and after each of the treatments. Note that pre-treatment with PMF90 significantly attenuated the overall changes in voiding behavior induced by CYP treatment. (**A**) At 24 and 36 h post-CYP, the mean voided volume per void of the vehicle-treated group was significantly lower than in the PMF90-treated group that displayed values that were not different from those recorded at baseline. At 24 h post-CYP, the maximum voided volume per void (**B**) and urinary frequency per 12 h (**C**) recorded for animals in the PMF90 group was also not different from baseline values, whereas these values were significantly altered in the vehicle-treated group. Finally, as shown in (**D**), prophylaxis with PMF90 also prevented an early and significant CYP-induced decrease in total urine voided volume. All error bars indicate SEMs. Two-way repeated measures ANOVA with a Bonferroni post hoc test was performed; * *p* < 0.05, ** *p* < 0.01, comparison between vehicle-treated and PMF90-treated groups; ^#^ *p* < 0.05; ^##^ *p* < 0.01 vs. initial self-control (n = 3, each group).

To investigate the prophylactic effect of PMFs, we used PMF90 (NOB, 60%; tangeretin, 30%) and injected the mice subcutaneousely (50 mg/kg PMF90) 24 h before CYP [150 mg/kg intraperitoneal (i.p.)] administration. Analysis of voiding behavior with the automated voided stain on paper (aVSOP) method showed that PMF90 prophylaxis significantly attenuated the increase in urinary frequency and decreased the maximum voided volume in CYP mice at 24 h after CYP administration when compared to the untreated CYP mice (Figure 1A–C). Total voided urine volume was significantly higher in the PMF90-treated mice at 12 h after CYP administration, but no significant difference was observed at 24 h post-CYP administration (Figure 1D).

### 2.2. Prophylactic Administration of PMF90 Suppressed Bladder Inflammation in CYP-Treated Mice

**Figure 2:**
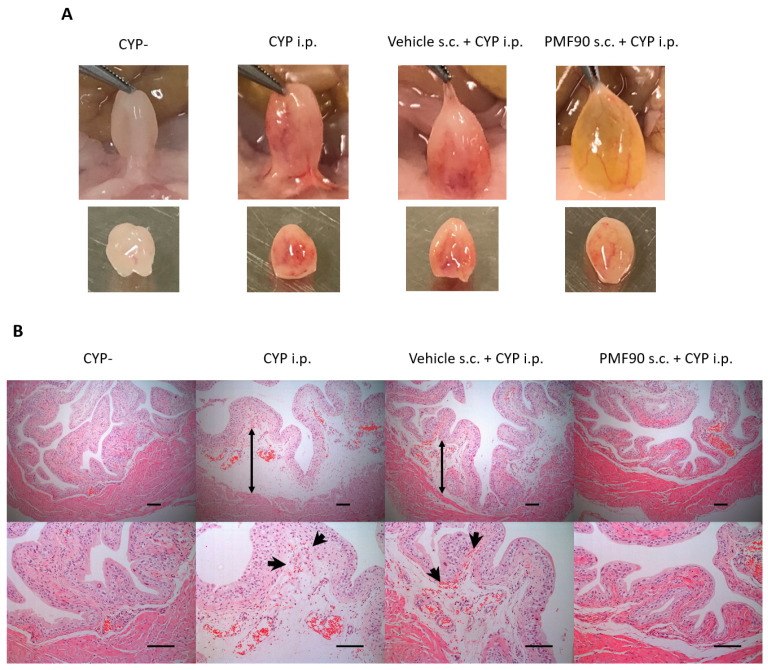
Macroscopic and microscopic comparison of bladders from control and cyclophosphamide (CYP)-treated mice and from vehicle and PFM90-pre-treated mice at 24 h following CYP administration. (**A**) Macroscopic view of the bladders. Note signs of erythema in the bladders of CYP mice and vehicle-treated CYP mice that are absent in the PMF90-treated CYP mice. (**B**) Histological hematoxylin and eosin (H&E) staining of bladder sections. Note the severe submucosal edemas (black arrows) and hemorrhages (black arrowheads) in CYP mice that were attenuated in PMF90- compared with vehicle-pre-treated mice. All scale bars indicate 100 µm.

On macroscopic examination, prophylaxis with PMF90 prevented the development of bladder erythema in CYP mice at 24 h after i.p. CYP administration (Figure 2A). Histological analysis of the bladder sections revealed that edema of the lamina propria and interstitial hemorrage in the lamina propria were supressed in the PMF90-treated CYP mice (Figure 2B).

### 2.3. Prophylactic PMF90 Administration Attenuated the Upregulation of Cx43 Expression and Activation of Inflammation-Related Genes in the Bladder Mucosa of CYP-Treated Mice

**Figure 3:**
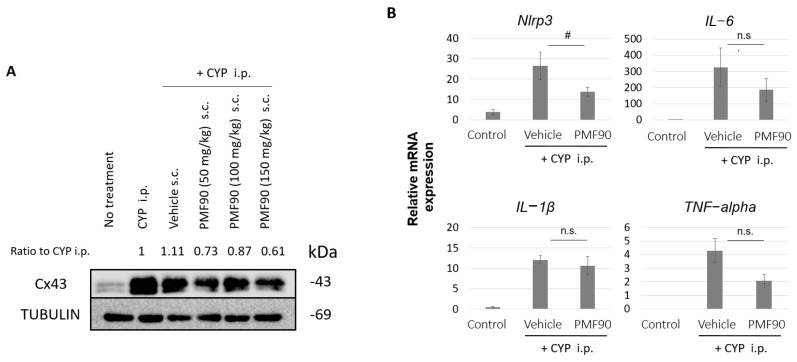
Effect of cyclophosphamide (CYP) treatment and of PMF90 pre-treatment on connexin 43 (Cx43) protein expression and inflammatory mediator mRNA expression in mouse bladder mucosa. (**A**) Cx43 expression in the bladder mucosa of a control (no treatment) mouse and mice at 24 h following CYP treatment which received no pre-treatment or were pre-treated with corn oil (vehicle) or with different doses of PMF90. Note the upregulation of Cx43 expression in the mucosa of CYP mice that was attenuated by administration of all the PMF90 doses at 24 h before CYP treatment. “Ratio to CYP i.p.” values presented above each lane correspond to Cx43/TUBLIN normalized by CYP i.p. lane. Cx43/TUBLIN values were determined from the densitometric analysis of protein bands using ImageJ software. One representative blot from two independent experiments with similar results is shown. (**B**) Expression of inflammation-related genes in the bladder mucosa of control (no treatment) and of vehicle (corn oil)- and PMF90 (50 mg/kg)-pre-treated CYP mice at 6 h after CYP administration. Note the significant increase in *IL-1β, IL-6, TNF-alpha*, and *Nlrp3* mRNA expression in the vehicle-pre-treated CYP mice when compared to controls and the effect of PMF90 pre-treatment in preventing *Nlrp3* upregulation and attenuating the increase in *IL-6* and *TNF-alpha* mRNA levels induced by CYP administration. The mRNA values were normalized by those of the housekeeping gene 18s ribosome. ^#^ *p* < 0.05 by Student’s *t*-test (n = 4, each group). n.s.; not significant difference. All error bars indicate SEMs.

We “peeled” the bladder mucosa (urothelium and lamina propria) from the detrusor to specifically investigate changes in the expression of Cx43 and key molecular mediators of inflammation in the mucosa of CYP mice and determine the effects of PMF90 on these molecular mediators. As shown in Figure 3A, at 24 h after CYP treatment, the expression of Cx43 protein in the mucosa was higher in CYP mice when compared to controls. Prophylaxis with PMF90 attenuated this CYP-induced upregulation of Cx43 in the mucosa (Figure 3A). Treatment with CYP also activated inflammation-related genes in the bladder mucosa. As shown in Figure 3B, at 6 h after CYP administration—a time point when *Cx43* mRNA expression was previously reported to be significantly increased in the bladder [28]—the mRNA expression of *IL-1β*, a major pro-inflammatory cytokine, was sigificantly upregulated in the mucosa of both CYP mice and PMF90-treated CYP mice when compared to controls. Expression of other pro-inflammatory cytokines, *IL-6* and *TNF-alpha*, was also signficantly increased in the mucosa following CYP administration. Upregulation of *IL-6* and *TNF-alpha* was attenuated in the PMF90-treated CYP mice, but not at a signficant level when compared to the untreated CYP group (Figure 3B). However, propylaxis with PMF90 prevented an increase of *Nlrp3*, a key component of the inflammasome, in the bladder mucosa of CYP mice (Figure 3B).

### 2.4. In Vitro Treatment with NOB Prevented IL-1β-Induced Upregulation of Cx43 Expression in Urothelial Cell Evaluation of Mechanosensitive Urothelial ATP Release in uCx43KO Mice

**Figure 4:**
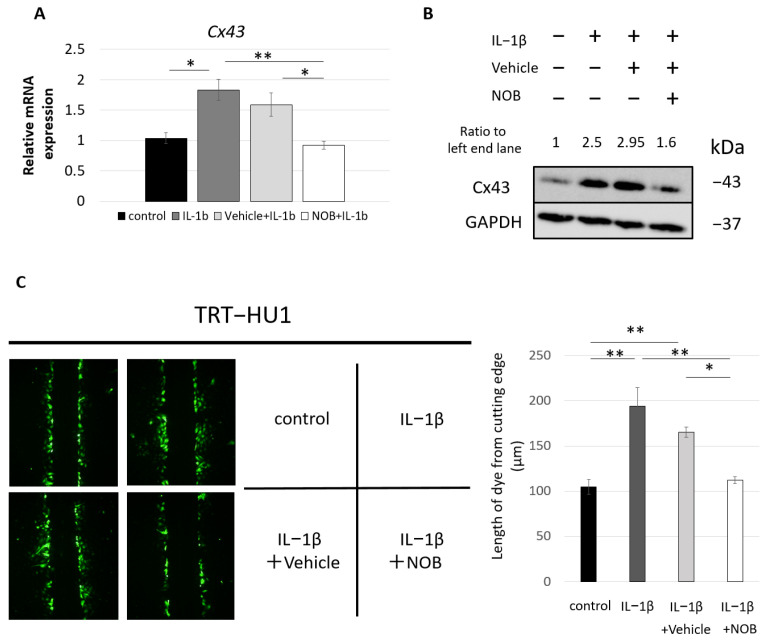
Effect of nobiletin (NOB) pre-treatment on IL-1β-induced increase in connexin 43 (Cx43) expression and gap junction function in urothelial cells. Immortalized human urothelial (TRT-HU1) cell cultures pre-treated with NOB or with DMSO (vehicle control) were stimulated with IL-1β (2 ng/mL) for 48 h. Cells were then harvested for Cx43 protein and mRNA quantification or were submitted to the scrape-loading–dye-transfer assay to assess changes in gap junction intercellular communication. Quantification of mRNA levels (**A**) and immunoblot (**B**) showing significant upregulation of Cx43 expression in TRT-HU1 cells following IL-1β stimulation, which was inhibited by pre-treatment of NOB. “Ratio to left end lane” values presented above each lane correspond to Cx43/GAPDH normalized by left end lane. Cx43/GAPDH values were determined from the densitometric analysis of protein bands using ImageJ software. The mRNA values were normalized by those of the housekeeping gene *GAPDH*. * *p* < 0.05 and ** *p* < 0.01 by one-way ANOVA with Tukey’s post hoc test (n = 3, each group). All error bars indicate SEMs. (**C**) IL-1β stimulation significantly increased the length of dye (lucifer yellow) spread from dye-loaded cells at the margin of the cut (“scrape”) to adjacent cells, when compared to the dye spread in control, non-stimulated cells. This finding of increased gap junction function correlates with the observed upregulation of Cx43 expression following IL-1β stimulation. Consistently, pre-treatment with NOB prevented the IL-1β-mediated increase in dye spread, i.e., increase in gap-junctional communication between TRT-HU1 cells. * *p* < 0.05 and ** *p* < 0.01 by one-way ANOVA with Tukey’s post hoc test (n = 8, each group). All error bars indicate SEMs.

To investigate whether the inhibitory effects of PMF90 on CYP-induced upregulation of Cx43 in the bladder mucosa involves mechanisms downstream of IL-1β signaling, we pre-administered NOB and determined its effect on IL-1β-induced Cx43 upregulation in hTERT-immortalized human urothelial (TRT-HU1) cells [35]. After stimulation with IL-1β (2 ng/mL) for 48 h, the mRNA expression of Cx43 was significantly increased when compared to unstimulated cells, and this response to IL-1β was suppressed by pre-administration of NOB (Figure 4A). A similar response was observed in relation to protein expression (Figure 4B). To evaluate the impact of altered Cx43 expression on gap junction function, we used scrape-loading and dye-transfer assays. As shown in Figure 4C, IL-1β increased gap junction coupling between TRT-HU1 cells, as evidenced by the significantly higher dye spread in the IL-1β-treated compared to control TRT-HU1 cultures. Pre-treatment with NOB prevented this effect of IL-1β stimulation in enhancing intercellular coupling in TRT-HU1 cultures. These results indicate that the IL-1β-induced increase in Cx43 expression is accompanied by a significant enhancement of gap junction function that is inhibited by NOB. They also suggest that the observed upregulation of Cx43 in the bladder mucosa of CYP mice is likely associated with the increased *IL-1β* expression and that PMF90 can act downstream in the IL-1β signaling pathway.

### 2.5. Effect of CYP Administration on Urothelium-Specific Cx43 KO Mice (uCx43KO Mice)

**Figure 5:**
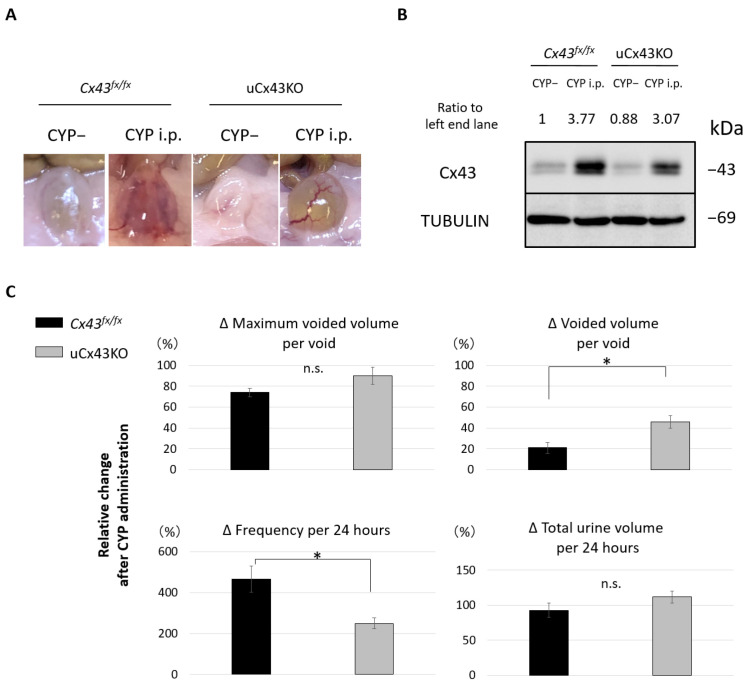
Development of bladder inflammation and urinary symptoms in the mouse model of cyclophosphamide (CYP)-induced cystitis is attenuated in the urothelium-specific connexin 43 knockout mice (uCx43KO mice). (**A**) Macroscopic in situ view of bladders from *Cx43^fx/fx^* mice (control) and uCx43KO mice at 24 h following treatment with CYP or saline (vehicle, CYP-). Note the signs of erythema and inflammation in the bladder of control *Cx43^fx/fx^* CYP mice that are virtually absent in the uCx43KO CYP mice. (**B**) Western blot showing Cx43 and tubulin (loading control) expression in the bladder mucosa (urothelium with lamina propria) at 24 h after CYP treatment. Note the marked increase in Cx43 expression in the *Cx43^fx/fx^* CYP mice when compared to the saline-treated control. Absence of urothelial Cx43 in the uCx43KO mice resulted in a reduced response to CYP, with Cx43 upregulation limited to its expression in the lamina propria. One representative blot from two independent experiments with similar results is shown. “Ratio to left end lane” values presented above each lane correspond to Cx43/TUBULIN normalized by left end lane. Cx43/TUBULIN values were determined from the densitometric analysis of protein bands using ImageJ software. (**C**) Comparison of the relative change in voided volume, frequency, total urine volume, and the maximum voided volume per void between *Cx43^fx/fx^* and u*Cx43*KO mice at 24 h following CYP administration. Note that compared to their saline-treated controls, the extent of change in voiding function experienced by uCx43KO mice following CYP treatment was significantly lower than that of Cx43^fx/fx^ mice regarding the voided volume per void and voiding frequency. * *p* < 0.05 by Student’s *t*-test (n = 3, each group). n.s.; not significant difference. All error bars indicate SEMs.

To assess the involvement of urothelial Cx43 in mechanisms of CYP-induced cystitis, we conducted studies with the urothelium-specific Cx43 KO mice (uCx43KO mice) [30]. On macroscopic examination, the development of bladder erythema at 24 h after CYP administration was attenuated in the uCx43KO CYP mice when compared to that in the Cx43^fx/fx^ CYP mice (Figure 5A). Immunoblotting analysis revealed that Cx43 expression in the mucosa of uCx43KO mice was increased following CYP administration but at levels that were somewhat lower than in Cx43^fx/fx^ mice (Figure 5B). This finding is consistent with the effects we observed of CYP treatment on wild-type mice and the low basal expression levels of Cx43 in the mucosa of uCx43KO that are attributed to its continued presence in the lamina propria. Analysis of voiding behavior, from 1 day prior to CYP administration to 2 days after CYP administration, showed that the relative changes in voided volume per void and in urinary frequency were significantly lower in the uCx43KO group when compared to those in the control Cx43^fx/fx^ group (Figure 5C). These results indicate that urothelial Cx43 participates in the development of bladder inflammation and urinary symptoms in the mouse model of CYP-induced cystitis and that with both its anti-inflammatory and preventive effect on Cx43 upregulation, PMF90 has great preventive potential.

## 3. Discussion

In the present study, prophylactic administration of PMF90 relieved the urinary symptoms in the mouse model of CYP-induced cystitis, suppressed the gross bladder inflammation, and attenuated the accompanied increase in Cx43 protein expression in the bladder mucosa. These protective effects involved the PMF90 anti-inflammatory action, targeting the activation of inflammation-related genes in the bladder mucosa, and its ability to prevent the upregulation of Cx43 driven by IL-1β signaling in urothelial cells, as indicated by the in vitro studies with NOB. This study also demonstrated the importance and the specific role played by urothelial Cx43 in the development of bladder inflammation and urinary symptoms in the mouse model of CYP-induced cystitis. Using the urothelium-specific Cx43KO mice, we showed that macroscopic signs of bladder inflammation and changes in voiding behavior following CYP administration were significantly attenuated in the absence of urothelial Cx43 expression. When combined, results from these in vivo studies with uCx43KO mice and in vitro studies with NOB in urothelial cell cultures suggest that through its inhibitory downstream effects on IL-1β signaling and urothelial Cx43 expression, PMFs can significantly attenuate bladder inflammation and ameliorate storage symptoms associated with inflammation.

NOB and tangeretin are the most abundant PMFs found in the peel of citrus fruits such as Shikuwasa and Ponkan and are known as natural bioactive substances with anti-inflammatory, anti-obesity, and anti-allergic properties. The anti-inflammatory effects of NOB have been reported, indeed, in the fields of infectious diseases, orthopedics, and neurology [36,37,38]. The mechanisms underlying the anti-inflammatory properties of NOB have been reported to involve inhibition of the NF-κB pathway and suppression of IL-6 and TNF-alpha signaling [36,37] or the AMPK activation and suppression of NLRP3, IL-1β, and IL-6 signaling [38]. NLRP3 is an important intracellular protein that forms inflammasomes and plays a role in immune response by mediating the secretion of pro-inflammatory cytokines, such as IL-1β in response to pathogen-derived signals (PAMPs) and to danger signals (DAMPs) released by damaged or dying cells, or in response to other stressors [39,40]. In the urological field, there are reports of NLRP3 involvement in prostatitis [41,42], in bladder denervation that develops after bladder outlet obstruction by benign prostatic hyperplasia, and in diabetic bladder dysfunction [43,44,45,46]. The role of NLRP3 in bladder inflammation has been recently reported in the lipopolysaccharide, protamine sulfate cystitis model and CYP-induced cystitis models and in the aging bladder [47,48,49,50].

The present study is in line with the proposed NLRP3 involvement in CYP-induced cystitis and suggests that the observed anti-inflammatory effect of PMF90 can be associated with the modulation of NLRP3 activity. In CYP-induced cystitis, activation of the NLRP3 inflammasome would occur in response to urothelial cell damage caused by acrolein, a CYP metabolite that is viewed as being responsible for the effects of CYP in the bladder. Acrolein is taken up by urothelial cells and its toxicity comes from the generation of reactive oxygen species, which, in turn, triggers downstream lipid peroxidation, protein oxidation, and DNA damage that ultimately results in necrotic cell death [51]. The importance of NLRP3 in mechanisms of CYP-induced cystitis was demonstrated in previous studies showing that inhibition of NLRP3 prevented increase in urinary IL-1β levels and reduced inflammation and bladder dysfunction in CYP-treated rats [47]. We have now shown that CYP induces upregulation of *nlrp3* expression in the bladder urothelium and that PMF90 prophylaxis inhibits this response (Figure 3). This effect of PMF90 on *nlrp3* expression is expected to translate into reduced NLRP3 inflammasome formation and consequent reduction of IL-1β production and release in response to urothelium damage. The process of IL-1β production is known to be regulated in two steps. That is, gene expression of IL-1β is induced by reactive oxygen species and other stimuli that induce transcription factor activity and promote the production of precursor IL-1β protein. It is then converted to mature IL-1β through processing via NLRP3 and other inflammasomes [39]. IL-1β expression was shown to be upregulated in the bladder of CYP-treated rats [52]. We observed that *IL-1β* expression was also increased in the urothelium of CYP mice and that this response was not prevented by PMF90 (Figure 3). The anti-inflammatory effect of PMF90, therefore, can be associated with the maturation process of IL-1β, though—and this marks a limitation—we did not measure urinary IL-1β to confirm reduced levels in PMF90-treated compared to untreated CYP mice.

The present study also indicates that the beneficial effects of PMF90 involve other mechanisms besides those associated with modulation of the NLRP3 inflammasome. We demonstrated that pre-administration of PMF attenuated the elevation of Cx43 in the urothelium of CYP-treated mice and inhibited in vitro the upregulation of Cx43 and accompanied increase of gap junction function induced by direct stimulation of human urothelial cells with IL-1β. There is increasing evidence that upregulation of Cx43 is involved in inflammation and the propagation of inflammatory signals via gap junctions in various cell types [28,53,54,55]. Zhang et al. revealed that AMPK activity attenuated the increase in Cx43 expression in a CYP-induced cystitis model focusing on the bladder smooth muscle layer and showed lesser urinary dysfunction in global Cx43 heterozygous mice compared with wild-type control mice [56]. Cx43 is expressed throughout the body, including inflammatory cells, so the observed beneficial effects granted by Cx43 heterozygosity are not expected to result solely from changes that occur at the smooth muscle layer. In the present study, using a urothelium-specific knockout (uCx43KO) mouse model, we actually demonstrated that urothelial Cx43 plays a major role in the development of bladder inflammation and urinary symptoms in the CYP-induced model of cystitis, focusing on the gap junction function. Since Cx43 functions not only in gap junctions but also in hemichannels, the anti-inflammatory effects granted by PMF modulation of Cx43 expression can be attributed to its action not only at the level of intercellular signaling mediated by gap junctions but also autocrine/paracrine urothelial signaling involving release of ATP and/or other messengers through Cx43 hemichannels [57,58]. Further research, however, is needed to evaluate the relative participation and role of urothelial Cx43 hemichannels in bladder inflammation.

In terms of translation and potential clinical application of the findings from this study, they support the urothelial Cx43 gap junction function and its inhibition as one of the targets and approaches to treat inflammatory bladder diseases. However, the ubiquitous expression of Cx43 throughout the body would make pharmacotherapy with gap junction inhibitors difficult. Glycyrrhetinic acid and its derivatives can block gap junction function and monoammonium glycyrrhizinate is a clinically available oral medicine for chronic hepatitis, dermatitis, or stomatitis [59]. One of the drawbacks of using glycyrrhetinic derivatives is that they tend to induce hypertension and hypokalemia, especially in the elderly, and have no indication for bladder inflammatory diseases [60]. To reduce side-effects and increase patient compliance, it would be ideal to directly deliver Cx43 inhibitors to the bladder by intravesical administration and use of target drug delivery systems, but these approaches have still to be explored and developed. In this regard, use of oral PMF could provide an alternative to modulate gap junction function. The safety of PMF has been confirmed by several clinical trials [16,61,62,63] and we are currently planning a clinical trial to evaluate its potential in preventing storage symptoms in inflammatory bladder conditions. Clinical applications in many other fields are also expected in the future.

## 4. Materials and Methods

### 4.1. Animals

Eight-week-old female C57BL/6 mice were purchased from CLEA Japan (Tokyo, Japan). The *Upk2Cre+* mice and the *Cx43^fx/fx^* mice were obtained from the Jackson Laboratory (Bar Harbor, ME, USA) and the bladder urothelium-specific Cx43 knockout (uCx43KO) female mice were generated by crossing *UPK2-Cre*+ and *Cx43^fx/fx^* mice, as described previously [30]. Mice were housed at constant room temperature with a cycle of 12 h light (7:00 to 19:00) and 12 h dark (7:00 to 19:00). Food and water were available ad libitum. This study was approved by the Kyoto University Animal Studies Committee (permit number: Medkyo19242), and all animals used in this study were treated according to the guidelines for animal experimentation of the experimental animal center of Kyoto University.

### 4.2. Mouse Model of CYP-Induced Cystitis

Mice were injected intraperitoneally with 150 mg/kg body weight of cyclophosphamide (CYP) diluted in 15 mL/kg body weight of saline, as previously described [28].

### 4.3. Treatment with PMF90

To assess the prophylactic effects of PMFs on CYP-induced cystitis, mice were injected subcutaneously with 50, 100, or 150 mg/kg body weight of PMF90 (60% nobiletin and 30% tangeretin) diluted in 2.5 mL/kg body weight of corn oil 24 h prior to CYP administration. Mice that were injected with vehicle alone (corn oil) served as controls.

### 4.4. Micturition Analysis

The automated voided stain on paper (aVSOP) method was used to evaluate voiding behavior, as described previously [31]. Briefly, animals were individually housed in the chamber of aVSOP machine in a sound-proof room with free access to food and water. After adapting to the environment for 2 days, voiding behavior was analyzed under 12 h light and 12 h dark conditions. In the PMF90 prophylaxis studies, the C57BL/6 female mice received 50 mg/kg of PMF90 or sham treatment with corn oil (control), administered subcutaneously. Twenty-four hours later, animals in both PMF90-treated and control groups received 150 mg/kg of CYP that was administered intraperitoneally. Voiding behavior before, after PMF90, and after CYP administration was recorded using the aVSOP method. In studies involving uCx43KO and *Cx43^fx/fx^* control female mice, voiding behavior was also recorded before and after CYP administration using the aVSOP method.

### 4.5. Tissue Harvesting

Mice from all experimental groups were anesthetized with isoflurane and euthanized by cervical dislocation at 24 h after CYP administration and the bladders were removed. The bladder mucosa (urothelium with lamina propria) and detrusor smooth muscle layers were then separated manually using fine forceps and immediately cryopreserved in liquid nitrogen for subsequent protein quantification for each mouse, or mixed with RNAlater (Sigma-Aldrich, St. Louis, MO, USA) and preserved at 4 °C for mRNA quantification in the independent experiments.

### 4.6. Hematoxylin and Eosin Staining of Mouse Bladder

Mice from all experimental groups were anesthetized with isoflurane and euthanized by cervical dislocation at 24 h after CYP administration. The bladders were removed, fixed in 10% neutral buffered formalin, and embedded in paraffin blocks. Serial sections (5 µm) were cut and submitted to routine hematoxylin and eosin (H&E) staining. Sections were then examined for presence of interstitial edema and interstitial hemorrhage.

### 4.7. Urothelial Cells and Cell Culture

The hTERT-immortalized human urothelial (TRT-HU1) cell line was used in this study, as a previously reported [32,35,64]. TRT-HU1 cells were maintained in DMEM (Sigma-Aldrich, Burlington, MA, USA) containing 2 mM L-glutamine and 110 mg/L sodium pyruvate supplemented with 15% FBS (Sigma-Aldrich, St. Louis, MO, USA), non-essential amino acids (Sigma-Aldrich, St. Louis, MO, USA), and 1.15 mM 1-thioglycerol [32].

### 4.8. IL-1 β Stimulation and Pre-Treatment of Urothelial Cells with Nobiletin

Confluent cultures of TRT-HU1 cells were stimulated for 48 h with 2 ng/mL IL-1β (Peprotech, Rocky Hill, NJ, USA) in urothelial medium containing 0.5% FBS. For the prophylactic experiments using NOB(Wako, Tokyo, Japan), 100 µM NOB and the vehicle DMSO(Sigma-Aldrich, St. Louis, MO, USA) were added 2 h prior to stimulation with IL-1β.

### 4.9. Scrape-Loading and Dye-Transfer Assay

This assay is broadly used to assess gap junction intercellular communication [65]. Briefly, 35 mm dishes with confluent TRT-HU1 cells were bathed in a solution containing the gap junction permeant dye lucifer yellow (LY: 2 mg/mL) (LO259, Sigma-Aldrich, St. Louis, MO, US) and a diamond cutter was used to make a cut through the monolayer in the center of the dish. Cells were then rinsed with phosphate-buffered saline (PBS; Wako Pure Chemical Industries, Osaka, Japan) 5 min after scraping and fixed with 4% paraformaldehyde. Dishes were then examined with an epifluorescence microscope, images of the cut line were taken, and the length of LY dye spread into the monolayer, from loaded cells on the cutting edge, was measured using ImageJ software (http://rsb.info.nih.gov/ij/, accessed 11 February 2020).

### 4.10. Real-Time Quantitative PCR (qPCR)

Total RNA was extracted from the mouse mucosa or TRT-HU1 cultured cells using RNeasy Mini kits (Qiagen, Hilden, Germany) according to the manufacturer’s protocols and complementary DNA was synthesized from 1 µg of RNA using ReverTra Ace qPCR RT Kit (TOYOBO, Osaka, Japan). The qPCR was performed with SYBR Green PCR Master Mix (Life Technologies, Carlsbad, CA, USA) and a 7300 Real-time PCR system (Life Technologies). The thermal cycling conditions were 94 °C for 15 s, 60 °C for 15 s, and 72 °C for 1 min. Values were adjusted relative to the expression levels of the housekeeping gene *Gapdh* or 18s ribosome. The primers used are listed in Supplementary Table S1. The ΔΔCt method was used to determine the relative expression levels of the genes of interest.

### 4.11. Immunoblotting

Bladder mucosa and TRT-HU1 cultured cells were lysed with the radioimmunoprecipitation assay (RIPA) buffer containing proteinase inhibitors. Total cellular protein concentrations were determined using detergent compatible (DC) protein assay reagent (Bio-Rad Laboratories, Richmond, CA, USA). Protein lysates (20 µg) were resolved by SDS-PAGE using 10% gel and transferred to polyvinylidene difluoride membranes (Millipore, Bedford, MA, USA) using a Mini Trans-Blot Cell system (Bio-Rad Laboratories). Membranes were blocked with 5% bovine serum albumin diluted in Tris-buffered saline with Tween^®^ 20 detergent (BSA/TBST) and incubated at 4 °C overnight with primary antibodies diluted in 1% BSA/TBST followed by incubation at room temperature for 1 h with horseradish peroxidase-conjugated secondary antibodies diluted in 1% BSA/TBST, and bands were detected by enhanced chemiluminescence (SuperSignal West Pico Chemiluminescent Substrate, Thermo Fisher Scientific, Waltham, MA, USA). Images were acquired with the LAS-4000 imaging system (Fujifilm Life Science, Tokyo, Japan). Anti-Cx43 (C6219, Sigma-Aldrich, St. Louis, MO, USA, 1:8000), anti-Tubulin (2144, Cell Signaling Technology, Danvers, MA, USA, 1:1000) and anti-GAPDH (GAPDH, 2118, Cell Signaling Technology, Danvers, MA, USA, 1:5000) were used as the primary antibodies. The levels of Cx43, TUBLIN (loading control), and GAPDH (loading control) protein expression were quantified using ImageJ software (National Institute of Health, Rockville Pike, MD, USA, http://rsb.info.nih.gov/ij/, accessed 20 March 2022, Figure 3, Figure 4 and Figure 5). Values were normalized to the respective loading control and were expressed relative to control in the second lane from left (Figure 3) or the left end lane (Figure 4 and Figure 5).

### 4.12. Drugs/Reagents

Cyclophosphamide (CYP) was purchased from LKT laboratories, Inc., (St. Paul, MN, USA). Corn oil was purchased from Sigma-Aldrich (St. Louis, MO, USA). Nobiletin (NOB) was purchased from Wako Pure Chemical Industries, Ltd. (Osaka, Japan) and high-purity PMF90 (60% nobiletin and 30% tangeretin as the main components, with sinensetin and isosinensetin as a minor component) powder was kindly provided by Okinawa Research Center Co., Ltd. (Okinawa, Japan).

### 4.13. Statistical Analysis

All data are expressed as the means ± SEMs. BellCurve for Excel (Social Survey Research Information Co., Ltd., Tokyo, Japan) was used for statistical analysis. Unpaired *t*-tests, one-way ANOVA with Tukey’s post hoc tests, and two-way repeated measures ANOVA with Bonferroni post hoc tests were performed when appropriate. *p* < 0.05 was regarded as statistically significant.

## Data Availability

Not applicable.

## Supplementary Materials

Supplementary materials can be found at: https://www.mdpi.com/article/10.3390/ijms23095037/s1.
