# Supplementary material for: Flavonoid Nobiletin Attenuates Cyclophosphamide-Induced Cystitis in Mice through Mechanisms That Involve Inhibition of IL-1β Induced Connexin 43 Upregulation and Gap Junction Communication in Urothelial Cells
Source: Int J Mol Sci. 2022 May 1;23(9):5037. doi: 10.3390/ijms23095037 (PMC9102543; doi:10.3390/ijms23095037)
Supplement: Supplementary file 1 [file ijms-23-05037-s001.zip › ijms-1701331-supplementary.pdf]

International Journal of Molecular Sciences

MDPI

Table S1. Primers for real-time quantitative PCR.

Species	Gene name		Primer sequences	Amplicon size (b.p.)
Human	*Cx43*	Forward	AGCAAAAGAGTGGTGCCCA	63
		Reverse	TTGTCAAGGAGTTTGCCTAAGG	
	GAPDH	Forward	GAAGGTGAAGGTCGGAGTC	226
		Reverse	GAAGATGGTGATGGGATTTC	
Mouse	*Nlrp3*	Forward	TGCTCTTCACTGCTATCAAGCCCT	85
		Reverse	ACAAGCCTTTGCTCCAGACCCTAT	
	TNF-alpha	Forward	CATCTTCTCAAAATTCGAGTGACAA	175
		Reverse	TGGGAGTAGACAAGGTACAACCC	
	IL-1β	Forward	CAGGCAGGCAGTATCACTCA	90
		Reverse	TGTCCTCATCCTGGAAGGTC	
	IL-6	Forward	CCGGAGAGGAGACTTCACAG	102
		Reverse	TCCACGATTTCCCAGAGAAC	
	Rn18S	Forward	ACTCAACACGGGAAACCTCA	123
		Reverse	AACCAGACAAATCGCTCCAC	
